# Immunotherapy for renal cell carcinoma: New therapeutic combinations and adverse event management strategies: A review

**DOI:** 10.1097/MD.0000000000038991

**Published:** 2024-07-26

**Authors:** Xiaohan Ma, Jibing Chen, Sheng Chen, Xuan Lan, Zengzhao Wei, Hongjun Gao, Encun Hou

**Affiliations:** a Graduate School, Guangxi University of Chinese Medicine, Nanning, Guangxi, China; b Ruikang Hospital, Guangxi University of Chinese Medicine, Nanning, Guangxi, China.

**Keywords:** adverse events, combination regimens, immune checkpoint inhibitor, new targets, renal cell carcinoma, tumor microenvironment

## Abstract

Immune checkpoint inhibitor (ICI) combinations, as well as ICIs combined with tyrosine kinase inhibitors, have considerable potential for renal cell carcinoma (RCC) treatment. Newer targeted medications, gut microbiome, nanomedicines, and cyclin-dependent kinase (CDK) inhibitors demonstrate significant potential in preventing side effects and resistance associated with RCC treatment. Most patients, including those demonstrating long-term treatment effects, eventually demonstrate cancer progression. Nevertheless, recent studies have further revealed RCC pathogenesis and many acquired drug resistance mechanisms, which together have led to the identification of promising therapeutic targets. In addition to having roles in metabolism, immunogenicity, and the immune response to tumors, CDK4 and CDK6 regulate the cell cycle. Targeting CDK4 and CDK6, either separately or in combination with already approved treatments, may improve therapeutic outcomes in patients with kidney cancer. Other novel drugs, including pegylated interleukin 10, colony-stimulating factor 1 receptor inhibitors, CD40 agonists, and C-X-C receptor 4 inhibitors affect the tumor microenvironment and cancer cell metabolism. Moreover, a triple ICI combination has been noted to be efficacious. In general, compared with sunitinib as a single-drug treatment, newer ICI combinations improve overall survival in patients with RCC. Future research on the prevention of adverse events and medication resistance related to newer therapies may aid in ensuring effective treatment outcomes among patients with RCC. This article aims to summarize innovative immunotherapy drug combinations for RCC treatment and the mechanisms of action, drug resistance, and treatment of adverse events associated with these combinations.

## 1. Introduction

Renal cell carcinoma (RCC) is responsible for 2% of all cancer diagnoses and mortality globally.^[1]^ Individuals with a mutation in the von Hippel-Lindau (VHL) tumor suppressor gene have a high risk of clear cell RCC (ccRCC), the most common RCC subtype; in 2% to 3% of the cases, ccRCC is hereditary.^[2]^ The second-most common RCC type is papillary RCC; it is further divided into types I and II, which are primarily associated with changes in the cellular mesenchymal–epithelial transition factor and the transcription factor NRF2-antioxidant response component, respectively.^[3,4]^ Finally, translocation RCC is associated with the fusion of the genes encoding the transcription factor E3 or EB, whereas chromophobe RCC is associated with changes in the genes encoding p53 and phosphatase.^[5,6]^

Despite considerable improvements in the management of advanced RCC over the last 10 years, most patients do not experience long-term clinical benefits. RCC is an immunogenic cancer, which has been historically treated with cytokines such as interleukin (IL) 2 and interferon α. Nevertheless, the current RCC treatment strategies mainly include the use of immune checkpoint inhibitors (ICIs) in combination with treatment modalities.^[7]^ In RCC patients at an advanced stage, tyrosine kinase inhibitors (TKIs) synergistically increase the efficacy of treatment with programmed death (PD) 1/PD ligand 1 (PD-L1) monoclonal antibody. Moreover, cytotoxic T-lymphocyte associated protein 4 (CTLA4), a protein associated with cytotoxic T cells, has a nonredundant effect in RCC treatment.^[8]^

Doublet combination therapy, typically involving 2 ICIs or an ICI and a TKI, can enhance clinical outcomes in patients with RCC.^[9]^ A meta-analysis of 6 phase III studies involving 5175 patients with primary metastatic RCC showed that immune-based combinations reduced the risk of death and more than tripled the complete response rate.^[10]^ On the basis of 6 independent clinical and laboratory risk variables, the International Metastatic Renal Cell Carcinoma Database Consortium (IMDC) criteria to classify RCC patient prognoses as favorable (0 risk factors), intermediate (1–2 risk factors), or poor (3–6 risk factors).^[11]^ This classification aids oncologists in recommending therapeutic options to their patients with RCC. Although the newer ICI-based therapies have demonstrated efficacy in advanced RCC treatment, some limitations, such as those related to the patient selection criteria, drug resistance mechanisms, and medications applicable for combination therapy, remain. The current review discusses immunotherapy-based combination regimens for primary therapy for advanced RCC, the supporting evidence, and the prospective novel combinations. A summary of effective methods for adverse reaction management is also included.

## 2. Tumor microenvironment in RCC

The revolution in RCC treatment by immunotherapy highlights the urgent need for prognostic and predictive biomarkers to enhance personalized treatment models, as many patients still lack durable responses. Therefore, recognizing the biomarkers of RCC and the associated tumor microenvironment (TME) is crucial.^[12]^

### 2.1. T cells

**Figure 1.**
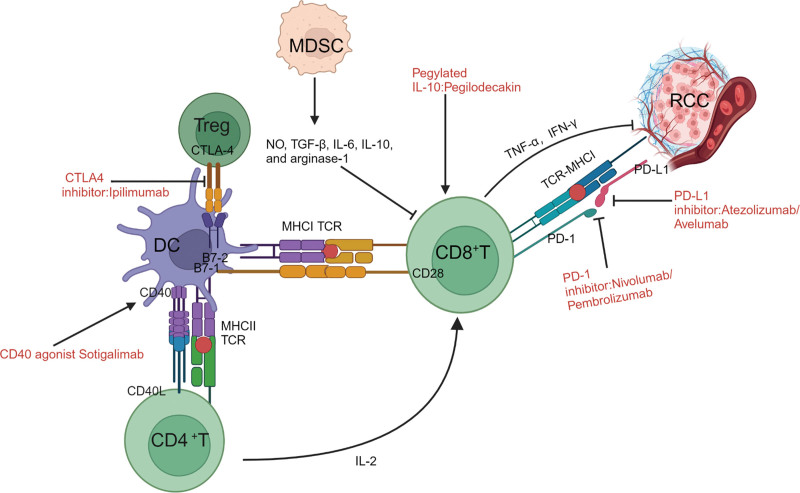
Synergistic effects of ICIs and agonists. DC deliver the MHCI complex to CD8+ T cells. DC surface-expressed B7 is a costimulatory molecule that binds to CD28 to activate CD8+ T cells. CD8+ T cells, recognize and destroy tumor cells by attaching their surface TCR to the MHC-I on the surface of the tumor cells. The CD40 agonistic antibody, a DC activator, improves antigen presentation and triggers CD8+ T cells when used against cancers that have weak immunogenicity. IL-10 inhibits inflammation and stimulates CD8+ T cells. PD-1/PD-L1/CTLA4 inhibitors block suppressor T cell signaling and reactivate the antitumor activity of CD8+ T cells. DC = dendritic cell.

The mechanisms by which CD8^+^ T cells target cancer cells include granzyme-mediated apoptosis, perforin-mediated apoptosis, and the Fas cellular surface death receptor–Fas cell surface death receptor ligand axis.^[13]^ Similarly, numerous immune cells are influenced by CD4+ T cells. The phenotype of CD4+ T cells determine their roles. For instance, type 1 T helper cells promote the activities of CD8+ T and B cells, which can destroy cancer cells directly by secreting INFs or tumor necrosis factors (TNFs). These cells also produce anti-inflammatory mediators, which prevent anticancer reactions.^[14]^ When PD-1 on activated T cells binds to the PD-L1 on RCC cells, activated T cells either die or lose their function. Dendritic cells that infiltrate tumors produce B7, another PD-1 ligand. Activated T cells may also express CTLA4, a negative feedback signal for T-cell activation at the lymph node level^[14]^ (Fig. 1).

### 2.2. Myeloid-derived suppressor cells

Myeloid-derived suppressor cells (MDSCs) are immature, very diverse myeloid cells produced by hematopoietic stem cells. In healthy individuals, MDSC levels are negligible. However, sustained stimulation under pathological conditions such as infection, trauma, and particularly, cancer frequently leads to defective variations of immature myeloid cells. In these situations, numerous immature cells, triggered by several inflammatory factors, accumulate in various organs and ultimately induce MDSC development. To suppress T and natural killer cell activities, MDSCs in the TME release cytokines such as nitric oxide, transforming growth factor (TGF)-β, IL-6, IL-10, and arginase-1. These cytokines interfere with dendritic cell function, preventing cancer cell antigen processing and delivery^[15–17]^ (Fig. 1).

### 2.3. von Hippel-Lindau

**Figure 2.**
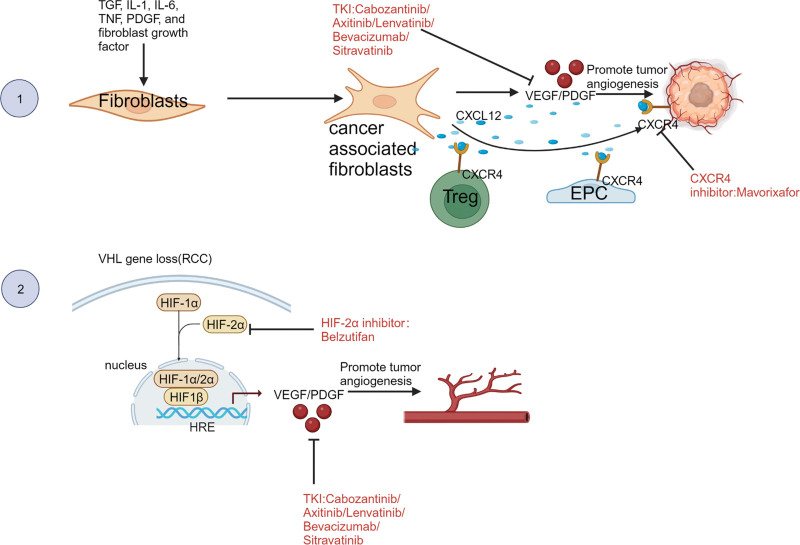
CXCL12/CXCR4 signaling pathways and VHL mutations. ① Through the action of CXCL12, CAFs facilitate angiogenesis by attracting endothelial progenitor cells (EPCs) to tumors. A CXCR4 inhibitor can reduce drug resistance in tumor cells, block angiogenesis and metastasis of tumor cells, and reduce the recruitment of immunosuppressive cells into the TME. ② As HIF1α/2α builds up, it forms heterodimers with HIF1β. These heterodimers go to the nucleus and attach to hypoxia response elements (HREs), which triggers HIF-mediated transcription and a pro-tumorigenic phenotype in cells. HIF-2α inhibitors reduce HIF-2α target gene transcription and expression that is associated with angiogenesis, tumor growth, and cell division. CXCL = C-X-C motif chemokine ligand, CXCR = C-X-C chemokine receptor.

When oxygen is available, VHL suppressor enzymes target hypoxia inducible factor (HIF)-1α and HIF-2α for proteasomal destruction. In most ccRCC patients who lack VHL, HIF-1α, and HIF-2α improve oxygen delivery through hypervascularization, thus promoting tumor formation.^[18,19]^ Drugs that target angiogenesis or vascular endothelial growth factor (VEGF) monoclonal antibodies are, therefore, the primary option for targeted therapy in RCC. However, TKIs have been noted to lead to immunotherapy resistance in ccRCC patients^[20]^ (Fig. 2).

### 2.4. Cancer-associated fibroblasts

When tumor cells are in close proximity to each other, mesenchymal fibroblasts become activated into cancer-associated fibroblasts (CAFs), which secrete cytokines such as TGF-β1 and platelet-derived growth factor.^[21]^ CAF-generated cancer-promoting molecules may function as paracrine molecules, which attach to receptors on the surfaces of nearby cancer cells and accelerate tumor growth. Angiogenesis and immune cell recruitment to tumor locations are facilitated by hypoxic CAFs, which also drive the remodeling of epithelial–mesenchymal transition, as well as the production and release of many growth factors, cytokines, and chemokines.^[22]^ CAFs also have immunosuppressive effects in T and natural killer cells, aiding tumors in evading the immune system.^[23]^ By binding to cancer cells and excluding T cells via C-X-C motif chemokine ligand 12/C-X-C chemokine receptor 4 signaling, fibroblast-activating protein–positive CAFs promote immunological repression^[24]^ (Fig. 2).

### 2.5. Macrophages

**Figure 3.**
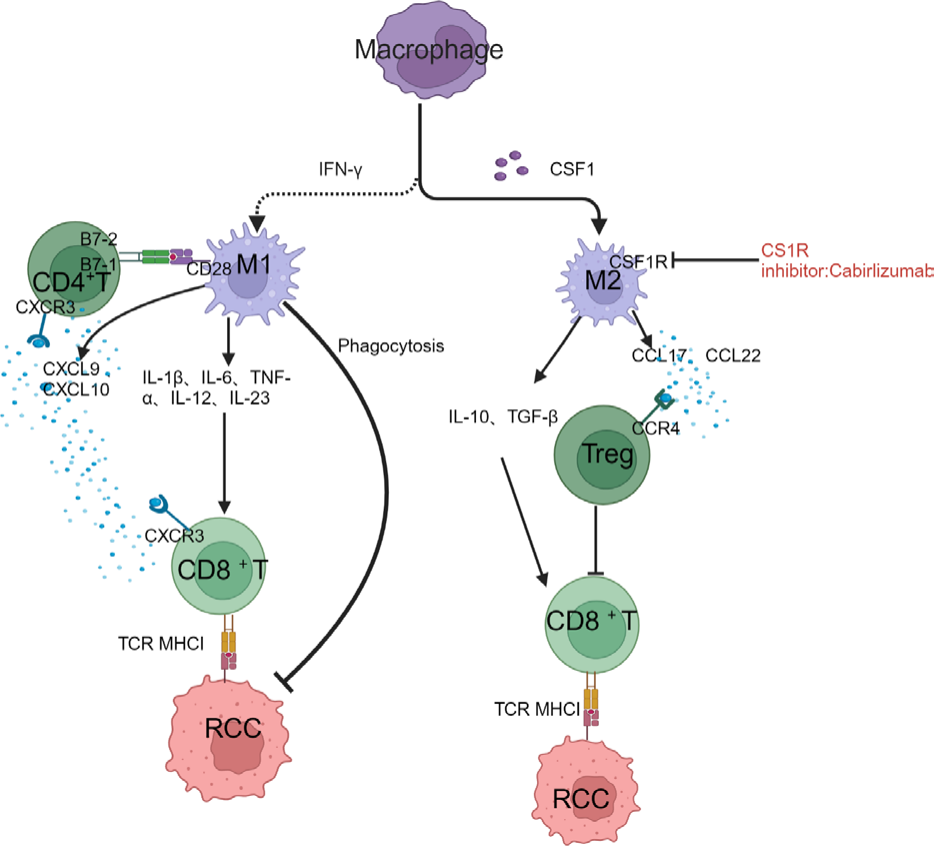
Macrophages and the CSF1/CSF1R pathway. Early M1 macrophages deliver antigens and facilitate CD8+ T cells by generating cytokines that promote inflammation. M2 macrophages inhibit CD8+ T cells and tumoricidal activity by releasing IL-10. They support the development of tumors by stimulating Tregs, which inhibit the immune system.

To deliver them to T cells, macrophages must first recognize and phagocytize antigens. Tumor-associated macrophages facilitate tumor progression and alter the tumor-supportive TME through the production of mediators such IL-1β, IL-6, TNF-α, C-C motif chemokine ligand 2, VEGF, platelet-derived growth factor, and TGF-β.^[25–27]^ Polarized macrophages with distinct functions can be divided into classically activated (M1) and alternatively activated (M2) macrophages.^[28]^ M1 macrophages are typically activated by interferon-γ, whereas M2 macrophages are alternately triggered by IL-10, IL-4, and IL-13.^[29]^ M2 and M1 macrophages are associated with tumor development and repression, respectively.^[30]^ Macrophage colony stimulating factor (CSF) 1, a chemotactic protein, interacts with its receptor CSF1R to promote monocyte tumor invasion and development into M2 macrophages^[30,31]^ (Fig. 3).

The relationship between drugs and TME is shown in Figures 1, 2, and 3. Created with BioRender.com.

## 3. Clinical trials on ICI-based combinations for RCC treatment

### 3.1. ICI–ICI combinations

In a clinical trial, patients with advanced RCC were randomly assigned in a 1:1 ratio to receive either nivolumab + ipilimumab or sunitinib. In patients with intermediate or poor prognosis risk, the coprimary outcomes were objective response rate (ORR), progression-free survival (PFS), and overall survival (OS). In the treatment group, secondary endpoints were ORR, PFS, OS, and adverse event (AE) frequency.^[32]^ In total, 550 and 546 patients received nivolumab + ipilimumab and sunitinib, respectively. Of all 1096 patients, 425 and 422 patients had intermediate and poor prognosis risk, respectively. Compared with sunitinib recipients, nivolumab + ipilimumab recipients demonstrated a higher ORR, as well as longer FPS and OS. In the favorable-risk group (n = 249), the 18-month OS rate demonstrated no significant difference between the sunitinib and nivolumab + ipilimumab recipients; however, the ORR and PFS were slightly higher in the sunitinib recipients than in the nivolumab + ipilimumab recipients. Compared with sunitinib, nivolumab + ipilimumab was reasonably well tolerated, with fewer grade 3 AEs (46% vs 63%). Moreover, treatment was terminated because of AEs in 22% and 12% of the nivolumab + ipilimumab and sunitinib recipients, respectively. Finally, 35% of the nivolumab + ipilimumab recipients experienced immune-mediated AEs; therefore, they were administered a high dose of glucocorticoids. Many of the nivolumab + ipilimumab recipients were alive at the 3-year follow-up; this therapeutic effect is expected to be sustained even after 5 years of treatment.^[33]^

### 3.2. ICI–TKI combinations

#### 3.2.1. Cabozantinib + nivolumab versus sunitinib

CheckMate 9ER compared the effects of cabozantinib + nivolumab and sunitinib in patients who had never received therapy.^[34]^ Based on their IMDC risk categories, only patients approved to receive cabozantinib + nivolumab were included. In every patient included with any of the IMDC risk ratings, PFS was the primary outcome. In total, 651 patients were enrolled, of whom 323 received cabozantinib + nivolumab and 328 received sunitinib. With a median follow-up of 18.1 months at the moment of the original analysis, cabozantinib + nivolumab outperformed sunitinib in the 12-month OS rate (85.7% vs 75.6%), PFS (16.6 vs 8.3 months), and ORR (55.7% vs 27.1%). Sunitinib led to a lower grade 3 AE rate than cabozantinib + nivolumab (70% vs 75.3%). Compared with the sunitinib group, the cabozantinib + nivolumab group had a higher rate of treatment termination due to any AE (16.9% vs 19.7%). At the 3-year follow-up, the median OS was longer in the cabozantinib + nivolumab recipients than in the sunitinib recipients (49.5 vs 35.5 months). In particular, regardless of the prognosis risk, cabozantinib + nivolumab produced more sustained treatment effects with higher complete response rates than sunitinib. Notably, the cabozantinib + nivolumab arm did not demonstrate safety issues during the follow-up. These findings provide additional evidence supporting the use of cabozantinib + nivolumab as the first-line therapy for advanced RCC.^[33]^

#### 3.2.2. Axitinib + pembrolizumab versus sunitinib

In a phase 3 clinical trial, patients with previously untreated advanced ccRCC were randomly assigned to receive either pembrolizumab (200 mg) intravenously every 3 weeks for up to 35 cycles along with axitinib (5 mg) orally twice daily (n = 432) or only sunitinib (50 mg) orally once daily for 4 weeks per 6-week cycle (n = 429). The primary outcomes were OS and PFS, whereas the secondary outcome was the ORR. Compared with sunitinib recipients, axitinib + pembrolizumab recipients had a higher 12-month OS rate and ORR, as well as longer PFS, at the beginning of the first analysis (median follow-up duration = 12.8 months). The grade 3 AE rate was higher in axitinib + pembrolizumab recipients than in sunitinib recipients (75.8% vs 70.6%); furthermore, more axitinib + pembrolizumab recipients than sunitinib recipients discontinued treatment due to AEs (rate of discontinuation due to AE = 13.9% for sunitinib alone, 30.5% for either axitinib or pembrolizumab, and 10.7% for both axitinib and pembrolizumab).^[35]^ Over the long term, axitinib + pembrolizumab was noted to be more effective than sunitinib, without any newer safety issues.^[36]^

#### 3.2.3. Lenvatinib + pembrolizumab versus lenvatinib + everolimus versus sunitinib

In a clinical trial, 1069 patients with advanced RCC were randomly assigned to receive lenvatinib + pembrolizumab (n = 355), lenvatinib + everolimus (n = 357), or sunitinib (n = 357). PFS was longer in recipients of lenvatinib + everolimus (median PFS = 14.7 months) and lenvatinib + pembrolizumab (median PFS = 23.9 months) than in sunitinib recipients (median PFS = 9. 2 months). Compared with sunitinib recipients, lenvatinib + pembrolizumab recipients but not lenvatinib + everolimus recipients demonstrated longer OS. The rates of grade 3 or higher AEs were 82.4%, 83.1%, and 71.8% in lenvatinib + pembrolizumab, lenvatinib + everolimus, and sunitinib recipients, respectively. In at least 10% of all patients, the grade 3 or higher AEs included hypertension, diarrhea, and elevated lipase levels.^[37]^ Finally, lenvatinib + pembrolizumab and lenvatinib + everolimus recipients had health-related quality of life scores comparable to or better than those of sunitinib recipients, specifically in terms of time to clinical deterioration. Therefore, the first-line treatment of lenvatinib + pembrolizumab might demonstrate favorable outcomes in patients with advanced RCC.^[38]^

In addition, a single-arm phase-2 trial confirmed that lenvatinib + pembrolizumab might be a safe option for first-line therapy in advanced non-ccRCC patients without a history of therapy.^[39]^

#### 3.2.4. Atezolizumab + bevacizumab versus sunitinib

IMmotion151, a phase 3 randomized clinical trial, compared the effectiveness and safety of atezolizumab + bevacizumab with those of sunitinib alone in patients with previously untreated metastatic RCC. Because of the manner in which it binds to VEGF, bevacizumab has been approved for use in conjunction with INF-α for metastatic RCC treatment.^[40]^ The trial enrolled 915 patients. Of them, 454 received sunitinib and 461 received atezolizumab + bevacizumab. Of the 915 patients, 362 (40%) were PD-L1 positive. At the first PFS evaluation and interim OS evaluation, the median follow-up was 15 months and 24 months, respectively. In the PD-L1-positive population, the median PFS was 11.2 and 7.7 months in atezolizumab + bevacizumab and sunitinib recipients, respectively. Treatment for resolving treatment-related AEs (TRAEs) was administered to 40% and 54% of the atezolizumab + bevacizumab and sunitinib recipients, respectively, and the rates of grade 3 to 4 TRAEs (which necessitated treatment termination) were 5% and 8% in the atezolizumab + bevacizumab and sunitinib recipients, respectively.^[41]^ In the final analysis, both the intention-to-treat and PD-L1-positive populations demonstrated similar median OS after either receiving atezolizumab + bevacizumab (36.1 and 38.7, respectively) or sunitinib alone (35.3 vs 31.6 months, respectively), without any detrimental consequences. These results confirm the efficacy and safety of bevacizumab in some patients with advanced RCC.^[42]^

#### 3.2.5. Avelumab + axitinib versus sunitinib

A phase 3 clinical trial compared the effects of avelumab + axitinib and sunitinib in 873 patients with previously untreated advanced RCC over a 6-week cycle. Of them, 434 received axitinib (5 mg) orally once daily for 6 weeks + avelumab intravenously once every 2 weeks and 439 received only sunitinib (50 mg) orally once daily for 4 weeks. In the PD-L1-positive population, avelumab + axitinib led to a substantially longer median PFS than sunitinib alone (13.8 vs 7.2 months)^[43]^; the combination also led to nearly 4 times higher ORR.^[44]^

#### 3.2.6. Cabozantinib + nivolumab + ipilimumab

In COSMIC-313, 550 patients were randomized to the experimental (n = 276) and control (n = 274) groups; their probability of 12-month PFS was estimated to be 0.57 and 0.49, respectively. Moreover, the response and grade 3 to 4 AE rates were respectively 43% and 79% in the experimental group and 36% and 56% in the control group. The OS outcomes have yet to be reported. For patients with previously untreated advanced RCC and intermediate or poor prognosis risk, treatment with cabozantinib + nivolumab + ipilimumab significantly prolonged PFS compared with nivolumab + ipilimumab.^[45]^

## 4. Newer ICI–drug combinations for RCC treatment

### 4.1. HIF-2α inhibitor + TKI

A phase 2 study compared disease progression, intolerable toxicity, or patient withdrawal in patients administered belzutifan (120 mg) + cabozantinib (60 mg) orally once daily. In total, 117 eligible patients were included. Of them, 52 (44%) were recruited in cohort 2 and received at least 1 dose of belzutifan + cabozantinib; their median age was 63.0 years, and 38 (73%) and 14 (27%) of them were male and female, respectively. The median follow-up duration was 24.6 months. In the 52 belzutifan recipients, the ORR was 30.8% (n = 16), with 2% (n = 1) experiencing complete recovery and 29% (n = 15) experiencing partial recovery. Hypertension was the most frequent grade 3 to 4 AE, occurring in 14 (27%) patients, and 15 (29%) patients experienced severe TRAEs. Finally, 1 death was attributable to a TRAE (i.e., respiratory failure). In patients with previously treated ccRCC, belzutifan + cabozantinib leads to considerable anticancer effects. However, additional randomized clinical trials on belzutifan + a VEGFR-TKI are warranted.^[46]^

### 4.2. CD40 agonist + CSF1R inhibitor + PD-1 inhibitor

A phase 1 study employing a 3 + 3 dose-escalation design with the CSF1R inhibitor cabiralizumab and the CD40 agonist sotigalimab alone or with nivolumab. A set dosage of cabiralizumab with or without nivolumab was provided after sotigalimab administration every 2 weeks until the cancer progressed or the side effects became unbearable. In cancer patients resistant to PD-1/PD-L1 inhibitors, dual macrophage-polarizing treatment with or without nivolumab for the first time demonstrated safety and pharmacodynamic efficacy. However, optimizing the timing and frequency of dose for this combination is warranted.^[47]^

### 4.3. IL-10 + PD-1 inhibitor

In advanced solid tumors, pegilodecakin (i.e., pegylated IL-10) stimulates oligoclonal T-cell growth and possesses single-agent efficacy. RCC patients who have undergone a considerable amount of prior treatment may benefit from pegilodecakin + pembrolizumab or pegilodecakin + nivolumab.^[48]^

A phase 1/1b multicohort dosage escalation IVY trial included 353 RCC patients who received pegilodecakin + pembrolizumab or pegilodecakin + nivolumab. The median PFS in all cohorts receiving either pegilodecakin + pembrolizumab or pegilodecakin + nivolumab was 13.9 months; moreover, their 6-month PFS, 1-year OS, and 2-year OS probabilities were 60%, 76%, and 61%, respectively. In contrast, the median PFS in all cohorts receiving pegilodecakin monotherapy was 1.8 months; moreover, their 6-month PFS, 1-year OS, and 2-year OS probabilities were 25%, 50%, and 17, respectively. Both combos failed to reach the median OS. Pegilodecakin + pembrolizumab and pegilodecakin + nivolumab led to ORRs of 33% and 43%, respectively. The most prevalent grade 3 to 4 TRAEs were hypertriglyceridemia, anemia, and thrombocytopenia. Pegilodecakin + nivolumab leads to significant clinical effects in patients with pretreated RCC. However, the safety of pegilodecakin alone and in combination with nivolumab, a PD-1 inhibitor, is similar to that reported previously.^[49]^

### 4.4. CXCR4 inhibitor + PD-1 inhibitor

Patients with metastatic ccRCC unresponsive to nivolumab monotherapy were administered mavorixafor (400 mg) daily + nivolumab (240 mg) intravenously every 2 weeks. Nine patients with a median age of 65 years were enrolled. At baseline, 5 of them had a stable illness, whereas 4 had a progressive illness. All 4 patients with a progressive illness achieved partial response to the combination therapy, and in these patients, the best response was a stable illness (median duration = 6.7 months). One patient with a stable illness at baseline who had previously received nivolumab monotherapy demonstrated a partial response to combination therapy. Four patients discontinued treatment due to AEs. Two patients each demonstrated elevation in alanine and aspartate aminotransferase levels, and 1 patient each demonstrated autoimmune hepatitis, chronic renal disease, increased lipase levels, maculopapular rash, and mucosal inflammation—all of which are all grade 3 TRAEs. A significant increase in C-X-C motif chemokine ligand 9 levels noted after mavorixafor treatment may be associated with positive clinical outcomes.^[50]^

### 4.5. PD-1 inhibitor + TKI

A phase 1 to 2 clinical trial optimized the starting dosage of sitravatinib with a fixed dose of nivolumab for 42 immunotherapy-naïve patients with advanced ccRCC and investigated the interaction between the 2 drugs. At the end of the trial, 12 (28.5%) of these patients were still receiving the study medications. The median follow-up duration was 18.7 months. Regardless of the baseline sitravatinib dosage, the ORR of sitravatinib + nivolumab recipients was 35.7% (n = 15), with a disease control rate of 88.1% (n = 37). One (2.4%) patient who received 80 mg/day of sitravatinib initially experienced a complete response. The median PFS of sitravatinib + nivolumab recipients was 11.7 months. By the end of the study, 34 (80.1%) patients were alive; the median OS has not been reported thus far. In summary, sitravatinib + nivolumab may exhibit excellent clinical and immunological effects with acceptable toxicity in patients with advanced ccRCC resistant to previous antiangiogenic treatment.^[51]^

### 4.6. PD-L1 inhibitor + TKI

#### 4.6.1. Cabozantinib + atezolizumab

COSMIC-021 assessed the effects of cabozantinib (40 or 60 mg) + atezolizumab in patients with ccRCC (n = 536 and 533, respectively) and those of cabozantinib (40 mg) + atezolizumab in patients with non-ccRCC (n = 532). In the 40-mg ccRCC, 60-mg RCC, and 40-mg non-ccRCC groups, the median follow-up duration was 25.8, 15.3, and 13.3 months, respectively; the median PFS (exploratory endpoint) was 19.5, 15.1, and 9.5 months, respectively; the ORRs (complete response) were 53%, 58%, and 38%, respectively; the rates of grade 3 or higher TRAEs were 71%, 67%, and 38%, respectively; and the rates of treatment termination due to TRAEs were 15%, 6%, and 3%, respectively. No patient demonstrated grade 5 TRAEs. Therefore, in both patients with advanced ccRCC and non-ccRCC, cabozantinib + atezolizumab provides excellent clinical efficacy, tolerability, and disease control.^[52]^

### 4.7. Cyclin-dependent kinase inhibitor + TKI

Various tumorigenic processes eventually encourage reproduction by interacting with cyclin-dependent kinase (CDK) 4 or CDK6 complexes during the cell-cycle phase G1. Therefore, CDKs that trigger cell-cycle transformation may be major therapeutic targets.^[53]^ In ccRCC, TKI and CDK4/6 inhibitor combinations have also been investigated. Curcumin, extracted from turmeric, has demonstrated anticancer activities in several cancer cell types. Some of its effects are elicited through CDK4 inhibition.^[54,55]^ Moreover, adding curcumin enhances the ability of sunitinib to suppress the development of the ccRCC cell line 786-O.^[56]^ Wogonin, a naturally occurring chemical produced from *Scutellaria baicalensis* Georgi, can block the CDK4-Rb pathway in 786-O cells.^[57]^ Sunitinib resistance in 786-O cells can abolished by treatment with wogonin or palbociclib, indicating the applicability of CDK4 inhibitors in the treatment of TKI-resistant RCC.^[57]^

## 5. TRAEs related to combination treatment

### 5.1. IrAEs with ICIs

Similar to other cancer therapies including chemotherapy, radiation, and surgery, immunotherapeutic treatment approaches, such as those using ICIs, demonstrate negative drug reactions; these are called immune-related AEs (irAEs). IrAEs may be a result of alterations in immune system mechanisms, particularly the loss of autoimmune tolerance or an increase in antigen sensitivity, ultimately leading to autoimmune reactions.^[58]^ Healthy tissue damage occurs due to a nonspecific T-cell hyperactivation response, causing cross-reaction with normal tissues. This leads to an overproduction of CD4 T-helper cell cytokines and abnormal migration of cytolytic CD8 T-cells. Immune-checkpoint inhibitors stimulate a broad T-cell repertoire expansion, counteracting tumor growth but reducing self-tolerance and damaging healthy organs. IrAEs significantly impact many organs and organ systems, including the gastrointestinal system, the skin, the liver, endocrine glands, and the myocardium.^[59,60]^

In a study, nivolumab monotherapy was noted to be associated with treatment-related rash and diarrhea in 10% and 12% of advanced RCC patients, respectively; similarly, in CheckMate 214, these rates were 22% and 27%, respectively.^[32,61]^

Studies have also revealed that among all patients with cancer, those with RCC demonstrate the highest risk of immune-related acute kidney injury. This may be due to immune cell infiltration being the most prevalent in RCC. As such, ICI treatment may worsen the unfavorable immunological impact of RCC.^[62]^ Even though ICI-related acute kidney injury rarely occurs, combination treatments such as CTLA4 inhibitor + PD-1/PD-L1 inhibitor and ICI + chemotherapy increase this risk.^[63]^

The TRAEs of ICI on the heart are significantly underestimated, and they are worse than those of conventional chemotherapeutics, particularly anthracyclines.^[64]^ TRAEs can be severe and even fatal in patients with metastatic RCC; as such, early treatment initiation is essential in these patients.

### 5.2. ICI combined with TKI

Patients receiving ICI–TKI combinations and those receiving sunitinib alone demonstrated a similar relative risk. However, the ICI–TKI combinations were associated with higher risks of all grades of diarrhea, grade 3 or higher of aspartate and alanine transaminase level elevations, all graded of hypothyroidism, and grade 3 or higher of decrease in appetite. Therefore, when selecting ICI–TKI combinations for metastatic RCC treatment, TRAE risks should considered.^[65]^

We learned from small molecule TKIs that the drug’s efficacy, derived from inhibiting the specific molecular target, is not necessarily directly related to the dose used: increasing the dose does not necessarily increase the effect (as the target may already be fully inactivated), while toxicity certainly increases. Therefore, identifying the dose-limiting toxicity and MTD (the traditional method used to define the therapeutic window for chemotherapeutics) may not be the best approach to determine the drug’s effect on the target in vivo. With immunotherapy, we are likely dealing with an even more complex situation, involving an unknown mechanism linking dose, efficacy, and toxicity.^[66]^

## 6. IrAE management

### 6.1. General treatment principles

IrAEs are often mild and brief. However, in some cases, particularly when using combination therapies, they can be worse. Effective treatment of irAEs depends on early detection, close observation, and quick reaction. As such, the American Society of Clinical Oncology has developed practice recommendations for irAE management.^[67]^

Before, during, and after ICI treatment, patients and their caregivers should be provided with relevant and updated information regarding immunotherapies, their mode of action, and their possible irAEs. Newly discovered posttherapy symptoms should be scrutinized closely. In general, during treatment with ICIs, patients should be closely monitored for grade 1 irAEs, such as hematologic and cardiac irAEs; neurologic toxicities may also be assessed occasionally. For a majority of the grade 2 irAEs, ICIs should be discontinued; they may be restarted when symptoms, test results, or both indicate that the irAE levels are grade 1 or lower. IrAEs may be treated with corticosteroids (first dose, 0.5–1 mg/kg/day prednisone or similar). For grade 3 or higher irAEs, a high dosage of corticosteroids such as prednisone (1–2 mg/kg/day or equivalent dosage) is recommended, which may then taper off over at least 4 to 6 weeks. For certain irAEs, if the symptoms do not subside after 48 to 72 hours of high-dose corticosteroid treatment, infliximab may be prescribed. A rechallenge with ICIs may be administered when the irAE symptoms, test results, or both return to grade 1 or lower; however, this step should be applied cautiously, especially in patients with early-onset irAEs. In general, dose modifications are not advised. Patients with irAEs due to combination treatment with a CTLA4 antagonist may be provided PD-1/PD-L1 monotherapy after their irAEs grade returns to 1 or lower. With the exception of endocrinopathies (managed by hormone replacement), grade 4 or higher irAEs warrant complete termination of ICIs.

### 6.2. Potential new drugs and targets for IrAE management

Despite the unavailability of relevant, comprehensive, accurate evidence, infliximab (TNF-α inhibitor) is currently recommended for the treatment of ICI-related myocarditis that does not respond to steroid treatment. However, infliximab use is associated with heart failure.^[68]^ Rituximab (CD20 inhibitor) is used to manage steroid-refractory AEs and ICI-induced Ig-refractory neuro-related AEs. Rituximab also prevents ICI-induced reactivation of primary membranous nephropathy, maintaining normal kidney function and ensuring long-term anticancer efficacy of ICIs.^[69,70]^ In the treatment of irAEs resistant to steroid treatment, the use of tocilizumab (IL-6 receptor inhibitor) considerably lowers treatment costs than that of infliximab.^[71]^ A 2021 multicenter case study reported that tocilizumab could alleviate irAEs that reduce anticancer therapy efficacy in patients with various cancer types.^[72]^ Tocilizumab can also aid in treating cancer-related cachexia; it may also synergistically improve the therapeutic effects of ICIs.^[73]^

### 6.3. Other methods

A growing body of research has linked irAEs to the gut microbiome. Firmicutes, such as *Faecalibacterium*, have been noted to be more prevalent in the intestines of patients with ICI-related colitis, whereas Bacteroidetes have been noted to be more prevalent in the intestines of those without ICI-related colitis.^[74]^ As such, the gut microbiome may aid in predicting irAE risk.

Flora transplantation may be used to enhance intestinal bacterial stability and intestinal flora population to improve patient prognosis.^[75,76]^ According to the 2020 treatment guidelines of the National Comprehensive Cancer Network, fecal microbiota transplantation is a potential therapy for immunosuppressive medication–resistant colitis.^[77]^

Nanotechnology has enabled rapid advancements in biomedicine and improved cancer treatment effectiveness. To improve immune response and reduce irAEs, nanodrugs targeting specific tissues or cells can be used.^[78]^ These nanodrugs may also contain 2 or more medications with synergistic activities.^[79]^ By modifying their size and surface characteristics, nanodrugs can also modify the drugs’ pharmacokinetics.^[80]^ In recent years, several innovative nano-based approaches for immunotherapy and urologic malignancy detection have been reported. Nanoparticles typically have a small size (1–1000 nm), strong reactivity, and a delivery impact.^[81]^ Nanoparticles are highly useful in cancer immunotherapy because of their multifunctionality. Nanoparticles can be used for the codelivery of several medications directly to the tumor or cancer cell. Moreover, to increase immunogenic cell death, they can be used to provide a controlled thermal dosage, targeted therapy for hyperthermia, or photodynamic therapy. Furthermore, the polymer and nanoparticle surfaces may be covered with ligands to ease their binding to immunostimulatory receptors.

The major limitation of cancer immunotherapy remains the high levels of toxicity, which occurs systemically and is reflected through the activation of circulating lymphocytes. Therefore, quickly clearing immunotherapeutic drugs out of the body’s circulation is essential. When these drugs are combined with nanoparticles, they can be delivered to the cancer cells or tumors more quickly and stimulate the TME—all while minimizing their systemic exposure considerably. CFM 4.16 (a novel class of apoptosis inducer) and sorafenib, which induced tumor hypoxia. These nanoparticles increased anticancer cytotoxicity and M1 macrophage abundance but reversed drug resistance in patients with RCC.^[82]^

## 7. Conclusion

In the current immunotherapy landscape for advanced RCC, nivolumab monotherapy is recommended only after the failure of earlier antiangiogenic treatment, and nivolumab + ipilimumab is effective only in previously untreated advanced RCC patients with intermediate or low prognosis risk. Moreover, PD-1/PD-L1 inhibitors combined with anti-VEGF therapy may be a particularly attractive treatment option for patients who respond effectively to antiangiogenic TKI treatments. Current research is focused on identifying biomarkers useful as therapeutic targets or treatment response predictors. Promising target mechanisms under active investigation include HIF-based angiogenic mechanisms, gut microbiome, key molecules that affect cellular metabolism, immune surveillance, and the TME. Some of these targets may revolutionize RCC further. The mechanisms of action of ICIs are unique; they enhance the immune system responses, restore normal immunogenic clearance mechanisms, and enhance the capacity of cytotoxic T cells. Therefore, in several cancer types, immunotherapies can result in a long-term response, enabling long-term disease control.

Immunotherapy, however, is also associated with a new set of AEs characterized by a lack of presentation and an association with a myriad of health conditions; these irAEs have varying onset times, sometimes overlapping with other cancer-related disorders. They represent newer diagnostic challenges and require newer management strategies so as to increase the survival and quality of life of patients undergoing immunotherapy for RCC.

## Author contributions

**Software:** Sheng Chen.

**Supervision:** Sheng Chen, Encun Hou, Hongjun Gao, Jibing Chen.

**Validation:** Sheng Chen.

**Visualization:** Sheng Chen.

**Writing – original draft:** Xiaohan Ma, Zengzhao Wei, Sheng Chen, Hongjun Gao, Jibing Chen.

**Writing – review & editing:** Xiaohan Ma, Zengzhao Wei, Sheng Chen, Xuan Lan,Encun Hou, Hongjun Gao, Jibing Chen.
